# Regulation of Translation by Lysine Acetylation in Escherichia coli

**DOI:** 10.1128/mbio.01224-22

**Published:** 2022-05-23

**Authors:** Sarah C. Feid, Hanna E. Walukiewicz, Xiaoyi Wang, Ernesto S. Nakayasu, Christopher V. Rao, Alan J. Wolfe

**Affiliations:** a Department of Microbiology and Immunology, Stritch School of Medicine, Health Sciences Division, Loyola University Chicago, Maywood, Illinois, USA; b Department of Chemical and Biomolecular Engineering, University of Illinois at Urbana-Champaign, Urbana, Illinois, USA; c Core Facility Center, The First Affiliated Hospital of Nanjing Medical University, Nanjing, China; d Biological Sciences Division, Pacific Northwest National Laboratory, Richland, Washington, USA; University of Pittsburgh

**Keywords:** acetylation, metabolism, ribosomes, translation, polysomal profiling

## Abstract

*N*ε-lysine acetylation is a common posttranslational modification observed in diverse species of bacteria. Aside from a few central metabolic enzymes and transcription factors, little is known about how this posttranslational modification regulates protein activity. In this work, we investigated how lysine acetylation affects translation in Escherichia coli. In multiple species of bacteria, ribosomal proteins are highly acetylated at conserved lysine residues, suggesting that this modification may regulate translation. In support of this hypothesis, we found that the addition of either of the acetyl donors acetyl phosphate and acetyl-coenzyme A inhibits translation but not transcription using an E. coli cell-free system. Further investigations using *in vivo* assays revealed that acetylation does not appear to alter the rate of translation elongation but, rather, increases the proportions of dissociated 30S and 50S ribosomes, based on polysome profiles of mutants or growth conditions known to promote lysine acetylation. Furthermore, ribosomal proteins are more acetylated in the disassociated 30S and 50S ribosomal subunits than in the fully assembled 70S complex. The effect of acetylation is also growth rate dependent, with disassociation of the subunits being most pronounced during late-exponential and early-stationary-phase growth—the same growth phase where protein acetylation is greatest. Collectively, our data demonstrate that lysine acetylation inhibits translation, most likely by interfering with subunit association. These results have also uncovered a new mechanism for coupling translation to the metabolic state of the cell.

## INTRODUCTION

**FIG 1:**
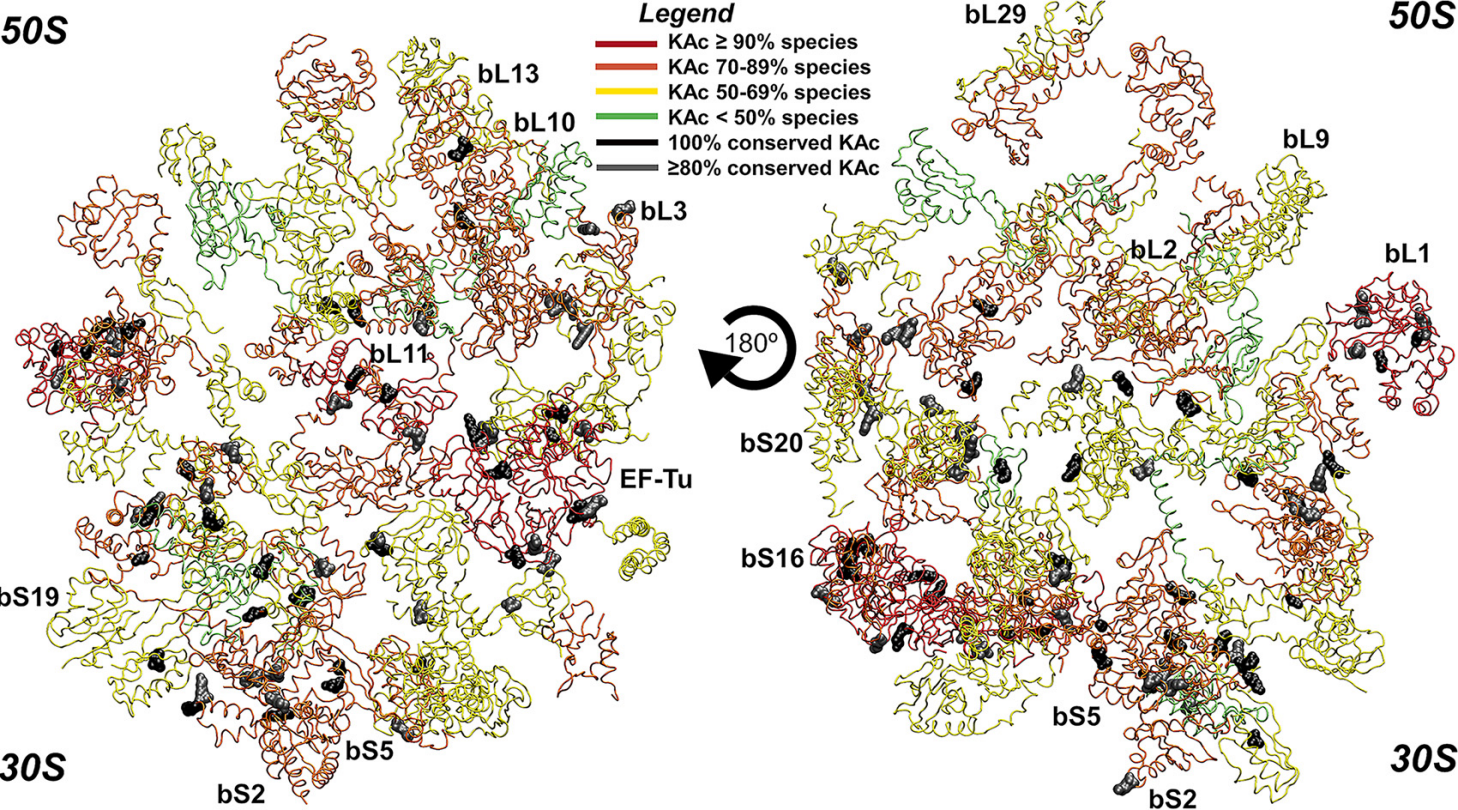
Conserved lysine acetylation sites on bacterial ribosomal proteins. Acetylation sites were extracted from a previously published proteomics data set of 48 phylogenetically distant bacteria (6). The degrees of acetylation across different species and conserved sites were mapped onto a ribosomal protein structure deposited in PDB (accession number 5UYK) using Visual Molecular Dynamics software version 1.9.3. Polypeptide chains are colored according to the percentage of species in which each ribosomal protein is acetylated, while the RNA molecules are hidden. Acetylations on conserved sites that were invariant or conserved in at least 80% of the 48 bacterial species are highlighted in black and gray, respectively.

*N*ε-lysine acetylation is a posttranslational modification found in all domains of life, and it is consistently observed in diverse bacterial species (1–3). This modification neutralizes the positive charge of lysine residues by covalently attaching an acetyl group to the amino group of the lysine side chain. While some acetylated lysines are known to alter protein activity, the vast majority remain uncharacterized (4, 5). One underinvestigated target of lysine acetylation is the bacterial ribosome, whose proteins are consistently acetylated at conserved sites in diverse bacteria (Fig. 1) (6, 7). Despite its being a common target of acetylation, little is known about the effect of acetylation on the ribosome and translation in general.

Lysines can be acetylated by two distinct mechanisms: enzymatically by lysine acetyltransferases using acetyl coenzyme A (acetyl-CoA) as the acetyl donor and nonenzymatically using acetyl phosphate or, more rarely, acetyl-CoA as the donor (8, 9). In Escherichia coli, Neisseria gonorrhoeae, and Bacillus subtilis, the majority of acetylations occur nonenzymatically (10–13). Some acetylations are removed by lysine deacetylases (14–16). Most bacteria express only one or two lysine deacetylases, which do not appear to act upon the majority of acetyllysines. Thus, most lysine acetylations are believed to be not reversed (17).

Proteins are principally acetylated when cells enter stationary phase during growth on excess carbon (7, 18, 19). To maintain flux through glycolysis, E. coli can ferment excess carbon to acetate through the Pta-AckA pathway, where phosphotransacetylase (Pta) converts acetyl-CoA and inorganic phosphate to acetyl phosphate and free CoA, and then acetate kinase (AckA) converts acetyl phosphate and ADP to acetate and ATP (20). More flux through this pathway increases the intracellular concentrations of acetyl phosphate, which is directly tied to the level of nonenzymatic acetylation in the cell (10, 13).

As cells enter stationary phase, the ribosomes undergo several changes. The rate of protein synthesis decreases, as does the rate of translation elongation (21). The number of 70S ribosomes decreases, either due to subunit dissociation or formation of 100S ribosomes, and the remaining 70S ribosomes become less active (22). Ribosome population differences that depend on a limiting nutrient suggest that metabolism regulates ribosome function; for example, phosphorus-limited E. coli cells maintain the same growth rate and protein levels as carbon- or nitrogen-limited E. coli cells but with fewer ribosomes (23). In support of a mechanism whereby acetylation regulates ribosome activity, recent work suggests that an accumulation of acetylations during stationary phase decreases the rate of elongation (24).

In this work, we investigated the effect of lysine acetylation on translation in E. coli. Using an *in vitro* transcription/translation assay, we found that acetyl donors inhibit translation but not transcription. To better understand the mechanism, we performed polysome profiling and found that fewer ribosomes form 70S complexes in high-acetylation mutants and/or under growth conditions known to promote acetylation. In contrast, under these conditions, we did not observe an acetylation-dependent effect on elongation rate. These results suggest that lysine acetylation inhibits translation by promoting disassociation or inhibiting association of the ribosome.

## RESULTS

### Acetyl donors inhibit translation.

**FIG 2:**
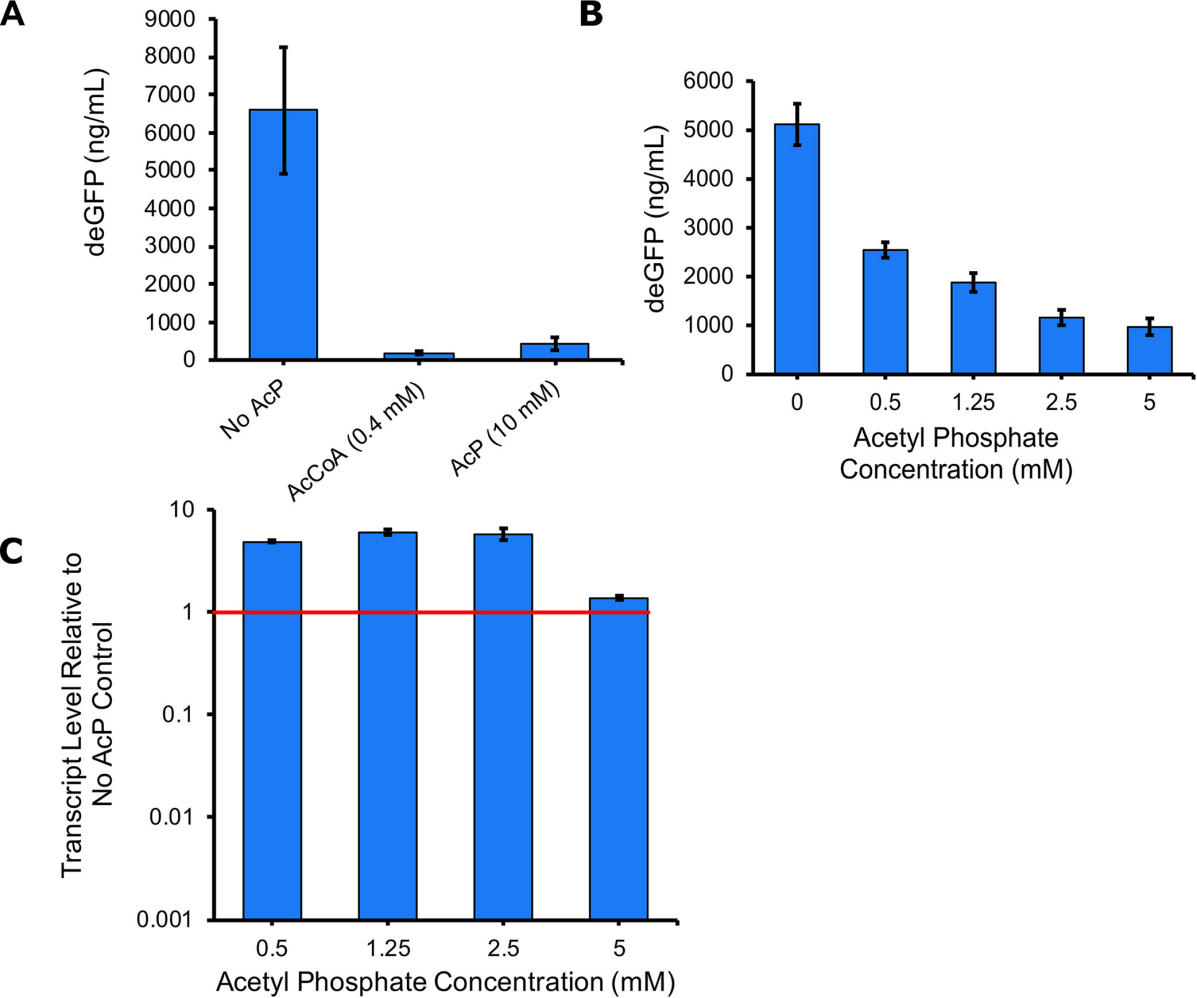
Addition of acetyl donors inhibits translation but not transcription. (A and B) deGFP synthesis by a cell-free transcription translation system was measured in the presence of acetyl-CoA or acetyl phosphate (A) and various concentrations of acetyl phosphate (B). (C) RNA was isolated from reaction mixtures for qRT-PCR. The expression of deGFP transcripts was determined relative to that in the no-acetyl phosphate control. Error bars represent the standard deviations of the results from two replicates.

Ribosomal proteins are highly acetylated, and the acetylated lysine residues are highly conserved in diverse species of bacteria (Fig. 1). Therefore, we hypothesized that ribosome acetylation would affect translation. To test this hypothesis, we used a cell-free transcription/translation system derived from E. coli cell lysates to measure the production of a green fluorescent protein (deGFP, a variant of enhanced green fluorescent protein [eGFP] optimized for cell-free synthesis) from a σ^70^-dependent promoter on a plasmid in the presence and absence of acetyl donor acetyl-CoA or acetyl phosphate (Fig. 2A) (25). The addition of either acetyl donor, at the upper range of its physiologically relevant concentration, strongly inhibited the production of deGFP as determined by fluorescence. While acetyl-CoA can nonenzymatically acetylate lysine residues, its contribution to *in vivo* acetylation is difficult to determine, as it essential in most organisms (7). Going forward, we chose to focus on acetyl phosphate, as it has been identified as the primary nonenzymatic acetyl donor in several bacteria (10–13). The concentration of acetyl phosphate varies based on the growth conditions and, in E. coli, can reach 5 mM (26). Using a spread of physiologically relevant acetyl phosphate concentrations, we found that deGFP production was inhibited in a dose-dependent manner (Fig. 2B). To determine whether the addition of acetyl phosphate was inhibiting transcription or translation, we used quantitative PCR to measure relative *degfp* mRNA levels. Consistent with a mechanism whereby acetyl phosphate inhibits translation, we found that mRNA levels did not decrease with increasing concentrations; at lower acetyl phosphate concentrations, mRNA increased relative to the amount in the untreated control (Fig. 2C). These results suggest that acetyl donors inhibit translation, likely by acetylating ribosomal proteins.

### Conditions promoting acetylation do not affect elongation.

**FIG 3:**
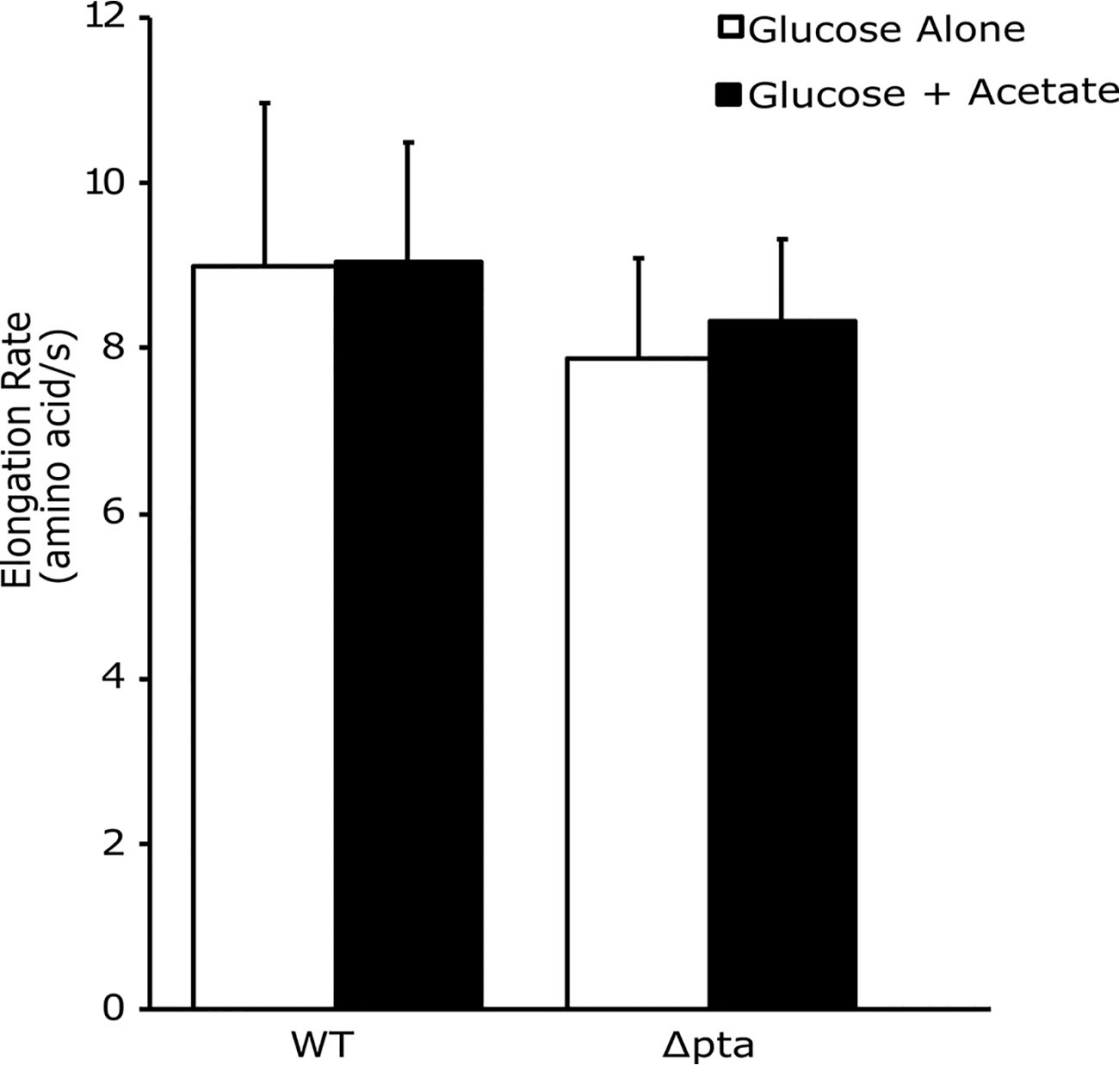
Conditions promoting acetylation do not affect elongation. Wild-type strain MG1655 (WT) and an isogenic Δ*pta* mutant were grown in MOPS with 0.2% glucose (white) or MOPS with 0.2% glucose supplemented with 0.27% acetate at 6 h (black). β-Galactosidase activity was induced at 8 h and used to calculate the elongation rate in amino acids/s. Error bars represent the standard deviations of the results from three replicates.

Previous work suggested that acetylation reduces the elongation rate (24). Such a mechanism could potentially explain the decreased rates of translation observed using the *in vitro* cell-free system. To test this hypothesis, we measured elongation rates using a LacZ induction assay (21, 27). To manipulate acetylation levels, or more precisely, acetyl phosphate concentrations, we grew a Δ*pta* mutant in MOPS (morpholinepropanesulfonic acid)-glucose minimal medium with or without acetate. This allowed us to manipulate the direction of the Pta-AckA pathway. When grown on MOPS-glucose, this mutant does not produce acetyl phosphate, as phosphate acetyltransferase (Pta) is needed to convert acetyl-CoA to acetyl phosphate, and thus, acetylation is low (20). When the growth medium is supplemented with sodium acetate, the Δ*pta* mutant accumulates acetyl phosphate, as acetate kinase (AckA) assimilates acetate to acetyl phosphate, and thus, acetylation is high (20). When *lacZ* was induced in stationary phase, we observed no difference in the elongation rate between the Δ*pta* mutant grown on 0.2% glucose versus its growth on 0.2% glucose supplemented with 0.27% sodium acetate (Fig. 3). The slight, nonsignificant decrease in elongation rate for the Δ*pta* mutant under both conditions is attributed to a modest growth defect of the Δ*pta* mutant relative to the growth of the wild type (Fig. S1). These results suggest that acetyl donors do not alter the rate of translation elongation, at least under the conditions tested in these experiments.

10.1128/mbio.01224-22.1 FIG S1 Growth in MOPS + 0.2% glucose with and without acetate supplementation over time. Wild-type MG1655 and an isogenic Δ*pta* mutant were grown in MOPS + 0.2% glucose or MOPS + 0.2% glucose supplemented with 0.27% acetate at 6 h (indicated by arrow). Optical density was measured at 600 nm. Each time point is the average of 3 biological replicates, with error bars representing the standard deviation. Download FIG S1, TIF file, 0.4 MB. Copyright © 2022 Feid et al. 2022 Feid et al. https://creativecommons.org/licenses/by/4.0/ This content is distributed under the terms of the Creative Commons Attribution 4.0 International license.

### High-acetylation mutants promote ribosome dissociation as determined by polysome profiling.

**FIG 4:**
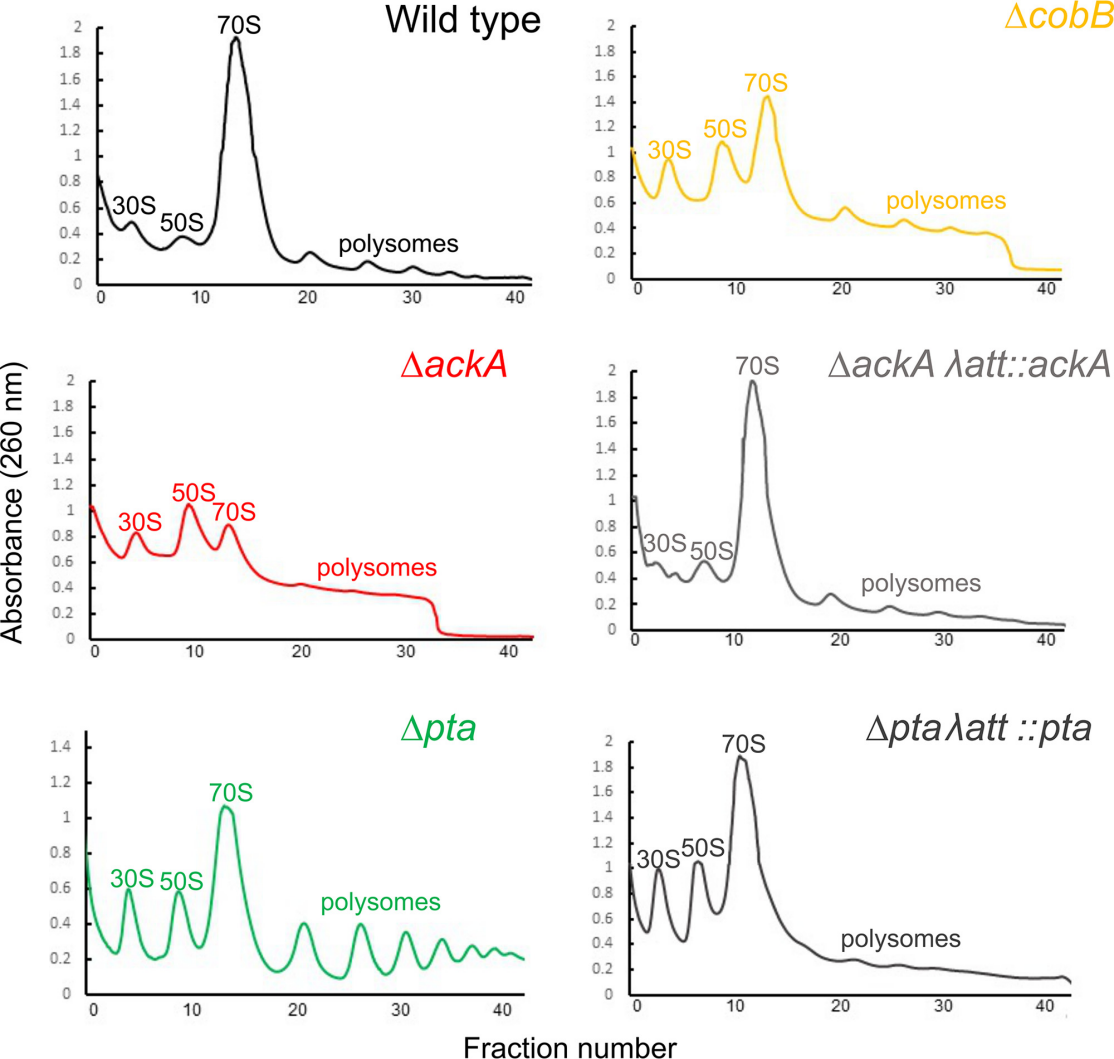
High-acetylation mutants promote ribosome dissociation. Polysome profiles of wild-type BW25113 and a series of isogenic mutants grown for 10 h in TB7 with 0.4% glucose are shown. 30S and 50S subunit peaks are marked, and 70S monosome peaks and polysome peaks are also marked. The identity of each peak was confirmed by RNA gel (Fig. 5A and data not shown).

**FIG 5:**
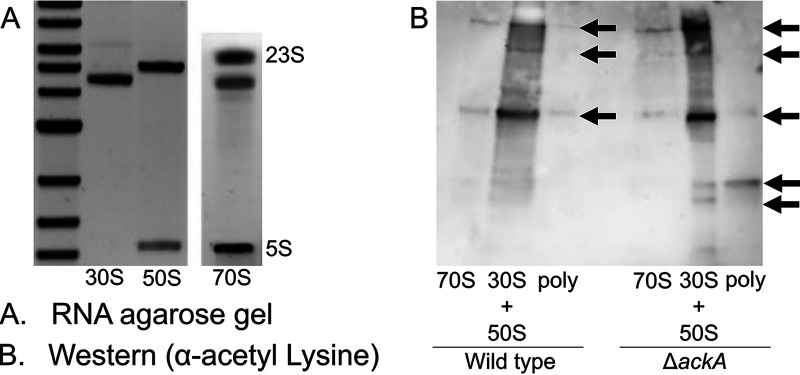
Analysis of polysomal gradient profiling fractions. (A) Agarose RNA gels for 30S, 50S, 70S, and polysomal peak fractions collected from the polysomal profile of wild-type BW25113 grown for 10 h in TB7 with 0.4% glucose. (B) Western blot using anti-acetylated-lysine protein antibody for 30S-plus-50S, 70S, and polysomal peak fractions collected from the polysomal profile of wild-type BW25113 and its isogenic Δ*ackA* mutant grown for 10 h in TB7 with 0.4% glucose. Loading was normalized to protein content. Arrows indicate bands of interest.

Our data suggest that acetyl donors, principally acetyl phosphate, inhibit translation in E. coli, most likely by acetylating ribosomal proteins. To gain further insight into the mechanism, we compared the polysome profiles for the wild type, a Δ*ackA* (high acetylation in a rich medium) mutant, and a Δ*pta* (low acetylation in a rich medium) mutant following 10 h of growth in TB7 (tryptone buffered to pH 7) containing 0.4% glucose (Fig. 4). A slight growth defect was observed in the Δ*ackA* and Δ*pta* mutants (Fig. S2). The wild-type profile exhibited a large peak associated with the 70S ribosome and smaller peaks associated with the 30S and 50S subunits. These peaks were verified using RNA gel electrophoresis (Fig. 5A). In contrast, the peak associated with the 70S ribosome was smaller in the Δ*ackA* (high-acetylation) mutant, comparable in size with the peaks associated with the 30S and 50S subunits (Fig. 4). The profile for the Δ*pta* (low-acetylation) mutant had larger 30S and 50S peaks and more polysomes than the wild type but was more like the wild type than the Δ*ackA* profile. As a control, we also profiled a complemented Δ*ackA* mutant (Δ*ackA* λ*att*::*ackA*) and a complemented Δ*pta* mutant (Δ*pta* λ*att*::*pta*). They exhibited polysome profiles identical to that of the wild type. These results suggest that conditions associated with high lysine acetylation favor dissociated subunits and that there is a more subtle effect associated with low acetylation.

10.1128/mbio.01224-22.2 FIG S2 Growth in TB7 + 0.4 glucose over time. Wild-type strain BW25113 and a series of isogenic mutants were grown for 16 h in TB7 + 0.4% glucose. Optical density was measured at 594 nm. Download FIG S2, TIF file, 0.4 MB. Copyright © 2022 Feid et al. 2022 Feid et al. https://creativecommons.org/licenses/by/4.0/ This content is distributed under the terms of the Creative Commons Attribution 4.0 International license.

### Proteins associated with the 30S and 50S ribosomal subunits are more acetylated than those associated with the 70S ribosomal complex.

The results discussed above demonstrate that mutants with highly acetylated proteomes have more disassociated ribosomes. This would suggest that the proteins within the dissociated 30S and 50S ribosomal subunits are more acetylated than those within the 70S ribosomal complex. To test this hypothesis, we performed Western blotting, using anti-acetyllysine antibodies, on the pooled 30S and 50S, the 70S, and the polysome fractions from the wild type and the Δ*ackA* mutant. For both strains, the 30S and 50S pooled fractions were more acetylated than the 70S or polysome fractions. This difference was more pronounced in the Δ*ackA* mutant, which also had a distinct band of acetylation in the polysome fraction not observed in the wild type. These results demonstrate that the disassociated 30S and 50S subunits contained more acetylated proteins than the 70S complexes (Fig. 5B).

### Growth on acetate promotes ribosome dissociation.

**FIG 6:**
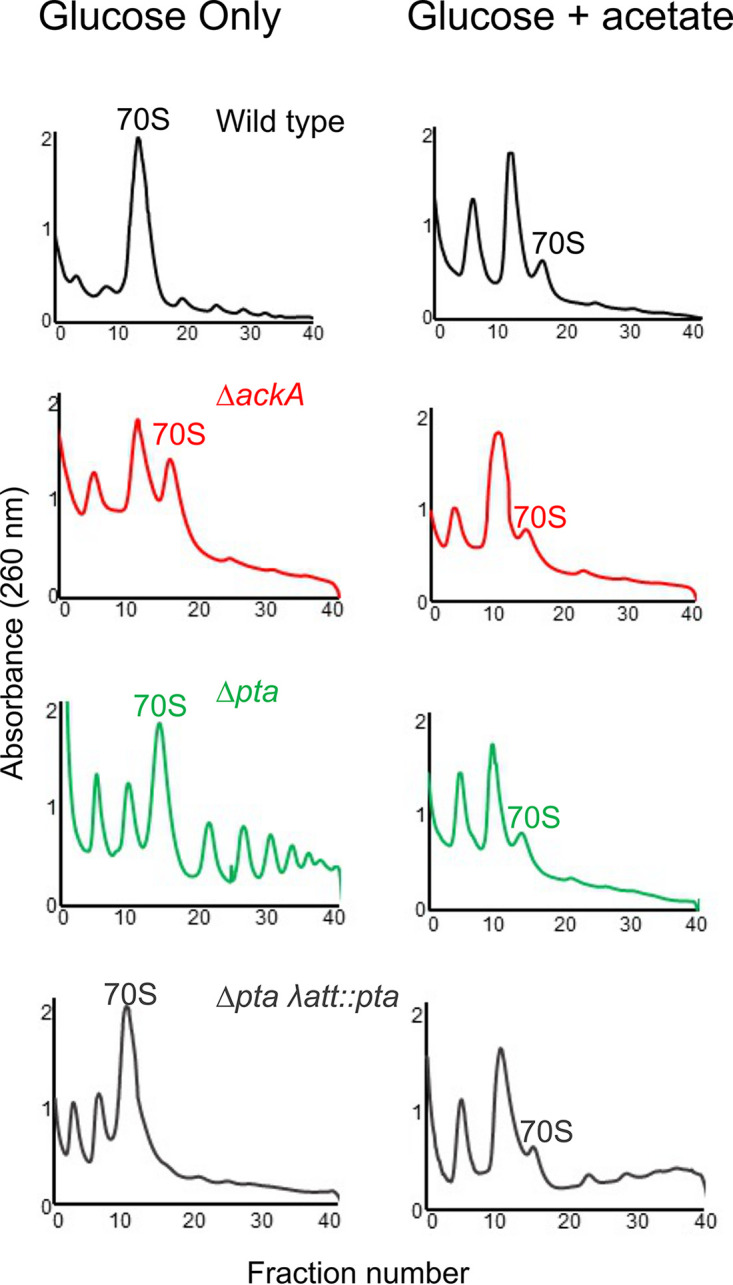
Growth on acetate promotes ribosome dissociation. Polysome profiles of wild-type BW25113 and a series of isogenic mutants grown for a total of 10 h in MOPS with 0.2% glucose or MOPS with 0.2% glucose supplemented with 0.27% acetate after 6 h are shown.

Because the growth conditions differed between our elongation and profiling results described above, we next performed polysome profiling for the wild type, the Δ*pta* mutant, and the Δ*ackA* mutant grown on 0.2% glucose versus 0.2% glucose supplemented with 0.27% sodium acetate (see Fig. S1 for an example of cell growth under these conditions). In further support of a mechanism whereby acetylation promotes dissociated ribosomes, we observed a reduction in the peak associated with the 70S ribosome and an increase in the peaks associated with the 30S and 50S subunits in both the wild type and the Δ*pta* mutant when acetate was added to the growth medium (Fig. 6). The Δ*ackA* mutant, which is already high in acetylation when grown in glucose, was less affected by the addition of acetate (Fig. 6). As growth on acetate is known to increase protein acetylation, these results further support the hypothesis that acetylation promotes ribosome dissociation (20). They also demonstrate that promotion of ribosome dissociation also occurs in the wild type and not just in high-acetylation mutants.

### Acetylation increases ribosome dissociation in wild-type E. coli beginning in late exponential phase.

**FIG 7:**
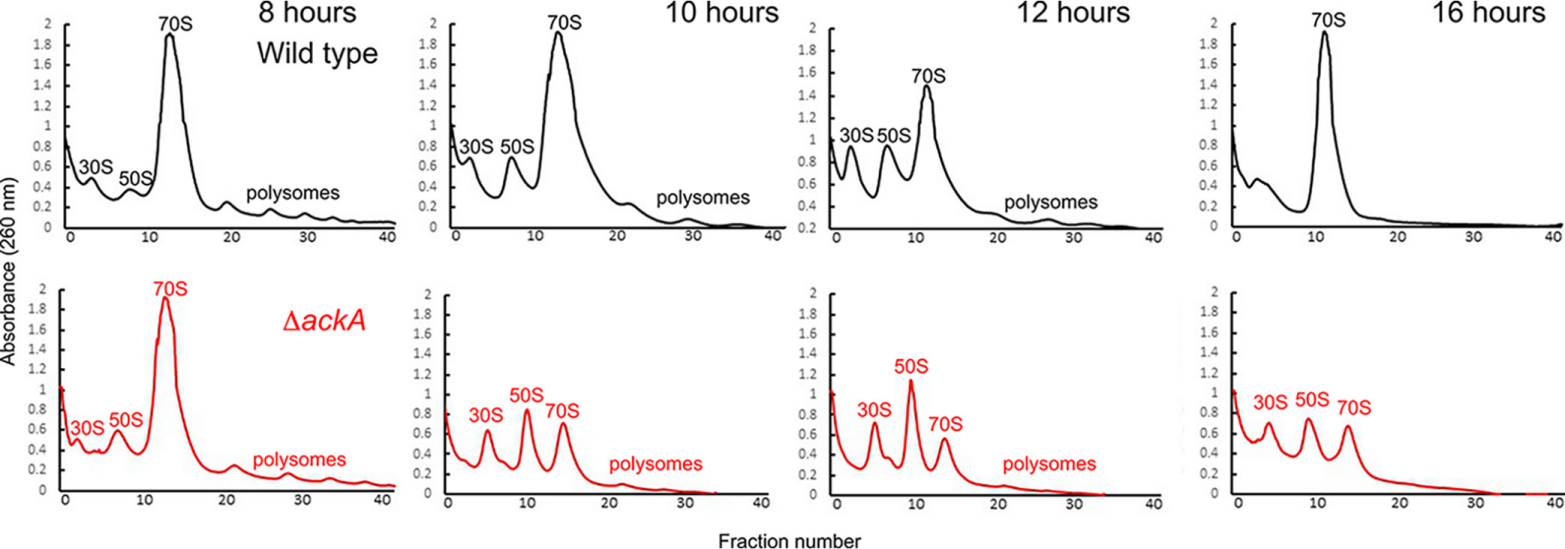
Polysomal gradient profiles for different time points. Polysome profiles of wild-type BW25113 and an isogenic Δ*ackA* mutant grown in TB7 with 0.4% glucose for the times noted are shown. The percentages of ribosomes in the 30S, 50S, and 70S fractions based on area under the curve calculations are provided in Table 1.

Lysine acetylations accumulate in E. coli cells during the transition into stationary phase. Therefore, we hypothesized that we would not observe any significant differences in the polysome profiles for the wild type and Δ*ackA* mutant during exponential-phase growth; rather, these differences would become significant only after entry into stationary phase. To test this hypothesis, we profiled the polysomes in the wild type and the Δ*ackA* mutant at multiple times along the growth curve (Fig. 7, Table 1). While there was little difference in the profiles at early time points, the profiles for the Δ*ackA* mutants diverged from the profile of the wild type at later time points, as the cells exited exponential growth (Fig. S2). In particular, the peak associated with the 70S ribosomal complex was reduced and the peaks for the 30S and 50S subunits increased. These differences persisted as the cells entered stationary phase.

**TABLE 1:** Portions of ribosomes in 30S, 50S, and 70S fractions over time

Time of growth (h)	Strain	% of ribosomes in indicated fraction		
		30S	50S	70S
8	Wild type	7.9	8.4	41.1
	Δ*ackA* mutant	10.1	6.7	48
10	Wild type	7	9.1	44
	Δ*ackA* mutant	18.5	18.2	21.9
12	Wild type	10.7	14.2	27.4
	Δ*ackA* mutant	13.7	24.8	17.1
16	Wild type	17.5		46.9
	Δ*ackA* mutant	21.2	17.7	18.5

Interestingly, we observed increased ribosome dissociation when the wild-type cultures entered early stationary phase. Not unlike the Δ*ackA* mutant, we observed larger peaks associated with the 30S and 50S subunits. However, when wild-type cultures entered stationary phase, the peaks associated with the 30S and 50S subunits decreased, suggesting that dissociation was a transient phenomenon associated with the transition to stationary phase. This dynamic was not observed in the Δ*ackA* mutant. Whether transient dissociation is due to acetylation is not known, although the Western blotting results indicated that dissociated ribosome subunits were more acetylated than the 70S complex.

## DISCUSSION

Ribosomal proteins are highly acetylated in diverse species of bacteria. A recent study suggested that acetylation of ribosomal proteins inhibits translation by reducing the rate of elongation (24). While our data suggest a more complex mechanism involving ribosome association/disassociation instead of elongation, both the previous and present studies demonstrate that acetylation affects translation. In our work, this was most clearly established when we measured protein production using a cell-free transcription/translation system. These experiments found that the addition of either of the acetyl donors acetyl phosphate and acetyl-CoA inhibits translation but not transcription in a dose-dependent manner. Our profiling experiments further demonstrate that fewer ribosomes form 70S complexes under conditions promoting high protein acetylation, either in mutants or by adding acetate to the growth medium. Moreover, the dissociated 30S and 50S subunits are more acetylated than the 70S complex, both in the wild type and the high-acetylation Δ*ackA* mutant. Taken together, these results demonstrate that ribosomal protein acetylation inhibits the ability of the ribosome subunits to form the 70S complex. Interestingly, we did not see any effect on translation elongation, which did not vary under conditions of high and low acetylation.

While the exact mechanism remains opaque, the simplest explanation is that acetylation inhibits translation initiation, where the 30S and 50S subunits associate with initiation factors, tRNA, and mRNA to form the 70S complex. Ribosomal lysine residues are important for subunit association, with four of the eight intersubunit bridges containing a lysine residue in E. coli. Among these interface residues, however, only K36 on the ribosomal protein L19 is known to be acetylated in E. coli (13, 17, 28). Other promising acetylation sites include three conserved lysine residues (K81, K84, and K100) on ribosomal protein L12, also known to be acetylated in E. coli. These conserved residues are located on a helix known to bind initiation factor 2 (IF2), elongation factors Tu (EF-Tu) and G (EF-G), and release factor 3 (RF3) (6, 29). IF2 is important for rapid subunit association during initiation (30–32). Mutations of K81 and K84 drastically impair subunit association, and altering the complementary charges between K84 of ribosomal protein L12 and D508 of IF2 impairs ribosomal subunit association (33). Because acetylation neutralizes the positive charge of lysine residues, it likely inhibits association by disrupting salt bridges along the subunit interfaces. While this mechanism is still speculative and requires further investigation to validate, it nonetheless provides one plausible explanation for how acetylation inhibits ribosomal assembly.

Proteins are most acetylated when the culture enters stationary phase (10, 13, 18). We observed a similar dynamic in our polysome profiling experiments. Indeed, subunit dissociation was most pronounced when cells were harvested from late-exponential/early-stationary-phase cultures, both in the wild type and in the high-acetylation Δ*ackA* mutant. One key difference was that disassociation occurred earlier in the Δ*ackA* mutant than in the wild type and, for the *ackA* mutant, continued throughout stationary phase. These results were expected, as the Δ*ackA* mutant accumulated acetyl phosphate due to the loss of acetate kinase. In other words, we expected that proteins would be more acetylated in the Δ*ackA* mutant even during exponential-phase growth, as shown in prior work (12, 18). The other key difference was that inhibition of ribosome association was transient in the wild type: subunit levels peaked in early stationary phase but then disappeared as the cultures fully entered stationary phase. In the Δ*ackA* mutant, on the other hand, dissociated subunits were observable well into stationary phase. Whether the latter differences are solely due to acetylation is not known, but the results clearly demonstrate that translation regulation by acetylation is a dynamic phenomenon that is growth phase specific.

E. coli produces acetate when the growth rate exceeds the respiratory capacity of the cell (20). When this occurs, the cells divert excess carbon flux toward acetate production. This enables cells to capture some energy that would otherwise be lost due to the inability to completely oxidize sugars at high rates. Acetate is also produced when carbon cannot be used to make biomass. This occurs when other essential nutrients/elements are depleted from the environment. For example, when nitrogen is depleted, the cells are unable to produce amino acids and instead divert excess carbon to acetate (34, 35). Under these conditions, protein acetylation is high (10).

Protein acetylation is tightly coupled to acetate metabolism in E. coli (7). This would suggest that acetylation of ribosomal proteins enables cells to couple translation and metabolism using a mechanism distinct from the stringent response. When a cell has a reduced need for protein synthesis, it has little need for ribosomes. This problem is partially addressed by the stringent response, where uncharged tRNAs induce the production of ppGpp(p) (36, 37). This, in turn, decreases transcription of the ribosomal operons. However, this mechanism only explains the rate of ribosome production. Because the cells cease to grow, existing ribosomes will not be “diluted away.” The cell, in other words, needs some mechanism for shutting down translation from preexisting ribosomes. Our hypothesis is that this is achieved, at least in part, through acetylation. Such a mechanism would explain why acetylation inhibits translation. Whether this mechanism is coupled with ribosome hibernation and the formation of the inactive 100S complex is presently unknown.

We conclude by addressing the question of elongation. As noted above, a previous study found that acetylation inhibits translation by reducing the rate of elongation (24). In the present study, we did not observe any significant changes in the elongation rate. Rather, our profiling experiments strongly suggest that acetylation inhibits the association of the 30S and 50S ribosomal subunits. One likely explanation for these discrepancies is that different growth conditions were employed. In our experiments, we grew wild-type and Δ*pta* mutant cells in minimal medium containing glucose in the presence or absence of acetate. The previous study compared elongation in the wild type and a Δ*pta* mutant during growth in minimal medium with acetate as the sole carbon source. In our experience, Δ*pta* mutants grow much more slowly than the wild type when acetate is the sole carbon source (20, 38). Whether decreased growth is due to the lack of acetylation or a by-product of growing a mutant defective in acetate metabolism on acetate is not known. Therefore, we supplemented the growth medium with glucose so that we could compare elongation in the wild type and the Δ*pta* mutant in the presence or absence of acetate. Further work will be necessary to resolve these discrepancies.

## MATERIALS AND METHODS

### Bacterial strains, media, and growth conditions.

All strains used in this work are derivatives of E. coli K-12 strain BW25113 or MG1655. Mutants were constructed by generalized transduction with P1kc, as described previously (39), with the Keio collection providing the appropriate deletion mutants (40), except for the Δ*ackA* λ*att*::*ackA* and Δ*pta*::*frt* λ*att*::*pta* complementation mutants. First, the *ackA* or *pta* gene was deleted using the method of Datsenko and Wanner (41). Then, Gibson assembly was used for plasmid construction to ligate the *ackA* gene (MG1655 genomic region 2411492 to 2412445) and the *pta* gene (MG1655 genomic region 2412769 to 2414943) containing their respective promoter regions with the pAH125 vector, according to manufacturer’s instructions. The constructed plasmids were integrated into the λ attachment site in the chromosome using the conditional-replication, integration, and modular (CRIM) plasmid method with the pINT-ts helper plasmid (42). The strains and plasmids used are detailed in Table 2.

**TABLE 2:** Bacterial strains and plasmids

Strain or plasmid	Description	Reference or source
Escherichia coli strains		
BW25113	F^−^ λ^−^ Δ(*araD-araB*)*567* Δ(*rhaD-rhaB*)*568* Δ*lacZ4787 rrnB3 rph-1 hsdR514*	41
MG1655	λ-*rph-1*	A. Ninfa, University of Michigan
AJW6217	BW25113 Δ*ackA*::*frt kn*	This study
AJW6267	BW25113 Δ*ackA*::*frt*	This study
AJW6215	BW25113 Δ*pta*::*frt kn*	This study
AJW6266	BW25113 Δ*pta*::*frt*	This study
HW3125	BW25113 Δ*ackA*::*frt* λ*att*::*ackA*	This study
HW3126	BW25113 Δ*pta*::*frt* λ*att*::*pta*	This study
AJW6341	MG1655 Δ*pta*::*frt kn*	This study
AJW6372	MG1655 Δ*pta*::*frt*	This study
AJW6377	MG1655 Δ*pta*::*frt* λ*att*::*pta*	This study
JW2293	Δ*ackA*::*frt kn*	40
JW2294	Δ*pta*::*frt kn*	40

Plasmids		
pINT-ts	Int_λ_	42
pAH125-ackA		This study
pAH125-pta		This study

Cells were cultured overnight in 5 mL lysogeny broth (LB) and subcultured in 250-mL flasks containing 50 mL TB7 (10 g/L tryptone buffered at pH 7.0 with 100 mM potassium phosphate), TB7 supplemented with 0.4% glucose, or MOPS (morpholinepropanesulfonic acid) minimal medium with 0.2% glucose as a carbon source for the times noted. When noted, MOPS cultures were supplemented with 0.27% acetate at 6 h. All cultures were grown at 37°C and aerated at 225 rpm with a flask-to-medium ratio of 5:1.

### Cell-free transcription/translation assay.

All experiments were performed using the myTXTL Sigma 70 master mix kit and P70a(2)-deGFP positive-control plasmid (Arbor Biosciences). Briefly, 15-μL reaction mixtures were prepared by combining 12 μL of master mix and plasmid and 3 μL acetyl phosphate or acetyl-CoA at the desired concentration. Distilled H_2_O was used for the control. Reaction mixtures were incubated for 2 h at 37°C in a heat block. Reactions were stopped on ice, and fluorescence measured. A standard GFP curve was used to calculate the amount of GFP synthesized.

### Quantitative PCR.

RNA was isolated from cell-free reaction mixtures using the MasterPure complete DNA and RNA isolation kit (Epicenter). After RNA isolation, cDNA was prepared using the iScript cDNA synthesis kit (Bio-Rad). A standard curve for reverse transcription-quantitative PCR (qRT-PCR) was prepared using E. coli strain B genomic DNA (gDNA), iTaq universal 2× SYBR green (Bio-Rad), and 16S primers (forward primer, CGGTGGAGCATGTGGTTTA, and reverse primer, GAAAACTTCCGTGGATGTCAAGA). Samples, no-template controls, and no-iScript controls were combined with iTaq universal 2× SYBR green (Bio-Rad) and primers for *degfp* (forward primer, GCACAAGCTGGAGTACAACTA, and reverse primer, TGTTGTGGCGGATCTTGAA). Reactions were carried out using the CFX Opus 96 real-time PCR system (Bio-Rad). The expression of *degfp* was calculated relative to its expression in the no-acetyl phosphate control.

### Elongation rate assay.

Translation elongation rates were measured using the LacZ induction assay (21, 27). Briefly, strains were grown overnight in MOPS with 0.2% glucose and subcultured to an optical density at 600 nm (OD_600_) of 0.1 in 50 mL of MOPS with 0.2% glucose. Cultures were incubated with shaking at 37°C until stationary phase. Acetate cultures were supplemented with 0.27% acetate at 6 h. At stationary phase, cultures were induced with 5 mM IPTG (isopropyl β-d-thiogalactopyranoside). Upon induction, at 30-s intervals, 100-μL amounts of culture were harvested into prechilled Eppendorf tubes containing 5 μL chloramphenicol (34 mg/mL) for 10 time points. Samples were snap-frozen and stored at −80°C prior to LacZ assay. The assay was largely adapted from the traditional Miller’s colorimetric method but utilized the fluorescent substrate MUG (4-methylumbelliferyl-d-galactopyranoside) instead of ONPG (*O*-nitrophenyl-β-d-galactopyranoside) (43, 44). Thawed samples were incubated with 400 μL Z buffer (60 mM Na_2_HPO_4_, 40 mM NaH_2_PO_4_H_2_O, 10 mM KCl, 2 mM MgSO_4_, 35 mM β-mercaptoethanol, pH 7) for 10 min at 37°C before 50 μL MUG (2 mg/mL) was added to each sample. Reaction mixtures were incubated at 37°C for 30 min and then stopped with 250 μL 1 M sodium carbonate. Fluorescence was measured in black-sided 96-well plates (excitation at 360 nm and emission at 460 nm). LacZ induction curves were made by plotting LacZ activity on the *y* axis and time postinduction on the *x* axis and further analyzed by square root plot to obtain the lag time for first LacZ molecule synthesis (*T*_first_) (45). LacZ is 1,024 amino acids in length, and the translation elongation rate is calculated as 1,024/*T*_first_.

### Polysome profiles.

E. coli cultures were grown overnight in LB and subcultured the next morning in 50 mL of TB7 with 0.4% glucose or MOPS minimal medium with 0.2% glucose to an OD_600_ of 0.02. Cultures were then grown at 37°C. When noted, cultures were supplemented with 0.27% sodium acetate at 6 h. At harvest time, 50 μL chloramphenicol (100 mg/mL) was added, and cultures were rapidly cooled and pelleted by centrifugation at 4,000 × *g* for 15 min at 4°C. Cells were then lysed in a buffer consisting of 10 mM Tris-HCl (pH 8.0), 10 mM MgCl_2_, and 1 mg/mL lysozyme by three freeze-thaw cycles. After the final freeze-thaw, 15 μL 10% sodium deoxycholate was added and cellular debris was pelleted by centrifugation at 9,400 × *g* for 10 min at 4°C. The supernatant was collected and stored at −20°C for profiling.

Profiles were run on a 10%-to-40% sucrose gradient prepared using a sucrose buffer consisting of 20 mM Tris-HCl (pH 7.8), 10 mM MgCl_2_, 100 mM NH_4_Cl, and 2 mM dithiothreitol (DTT). Gradients were prepared using the BioComp Gradient Master model 108. Each gradient was loaded with 300 L of E. coli lysate and spun using an SW-41 rotor in an ultracentrifuge at 175,117 × *g* for 3 h 45 min at 4°C. Gradients were fractionated using the ISCO/Brandel fractionation system by injecting a 50% sucrose solution below the gradient at 1.5 mL/min. Ribosomes were detected by the system’s UV spectrophotometer at 254 nm. Fractions were stored at −20°C for future analysis by Western blotting.

### RNA purification and electrophoresis.

Ribosome peak fractions were pooled. The 30S and 50S ribosomal peaks were processed directly, but the 70S ribosome fraction and polysome fractions were dissociated into 50S and 30S subunits, and bound mRNA was removed by loading each fraction onto a 10%-to-45% sucrose gradient made in disassociation buffer (20 mM HEPES, pH 7.5, 5 mM β-mercaptoethanol, 5 mM MgCl_2_, 50 mM NH_4_Cl, 0.1 mM phenylmethylsulfonyl fluoride [PMSF]). The gradient was centrifuged as described above, using an SW-41 rotor in an ultracentrifuge at 175,117 × *g* for 3 h 45 min at 4°C. Gradients were fractionated using the ISCO/Brandel fractionation system. To all individual pooled ribosomal fractions, a 1.5× volume of TRIzol reagent (Invitrogen) was added. Sample tubes were shaken for 15 s and incubated at room temperature for 10 min. Samples were then layered onto a Direct-zol RNA miniprep (Zymo Research) spin column, and RNA was extracted according to the manufacturer’s directions. Polysomal RNA can be isolated using TRIzol alone, but we were able to obtain cleaner RNA using the Direct-zol RNA miniprep (Zymo Research) in addition to TRIzol (Invitrogen). To visualize RNA, 0.5 μg of the purified RNA was mixed with 1.5× volume of deionized formaldehyde and RNA loading buffer (0.25% bromophenol blue, 0.25% xylene cyanol, 30% glycerol) and loaded onto a 1.2% agarose gel made in TBE (45 mM Tris-borate, 1 mM EDTA, pH 8.0) using diethyl pyrocarbonate (DEPC)-treated water. The gel was run for 45 min at 100 V, and RNA bands were visualized using SYBR green II RNA gel stain (ThermoFisher Scientific).

### Antiacetyllysine antibody Western blotting.

The protein concentrations within the fractionated sample loaded onto the gel were normalized by total protein content using the bicinchoninic acid (BCA) assay (Thermo Scientific Pierce, Waltham, MA). Proteins were separated by 12% sodium dodecyl sulfate–polyacrylamide gel electrophoresis (SDS-PAGE). Gels were rinsed in transfer buffer (25 mM Tris, 192 mM glycine, 10% methanol), and the proteins transferred onto a nitrocellulose membrane in transfer buffer for 1.5 h at 100 V at 4°C. After transfer, membranes were blocked with 5% milk in PBST (137 mM NaCl, 2.7 mM KCl, 10 mM Na_2_HPO_4_, 1.8 mM KH_2_PO_4_, 0.1% Tween) for 1 h and washed with PBST four times for 5 min each time. Primary rabbit antiacetyllysine antibody (Cell Signaling, Danvers, MA) was diluted 1,000-fold in 5% bovine serum albumin (BSA), added to the membranes, and incubated in the cold room with shaking. The membrane was washed 4 times with PBST for 5 min each time and incubated for 1 h in the dark at room temperature with anti-rabbit IgG horseradish peroxidase (HRP)-linked secondary antibody (Cell Signaling, Danvers, MA) diluted 2,000-fold in 5% milk. The membrane was washed 4 times with PBST for 5 min each time, incubated in ECL blotting substrate (Abcam), and imaged in the Protein Simple machine (Bio-Techne) (13, 19).

### Data availability.

Data available upon request.
