# Supplementary figures and images for: Regulation of Translation by Lysine Acetylation in Escherichia coli
Source: mBio. 2022 May 23;13(3):e01224-22. doi: 10.1128/mbio.01224-22 (PMC9239087; doi:10.1128/mbio.01224-22)

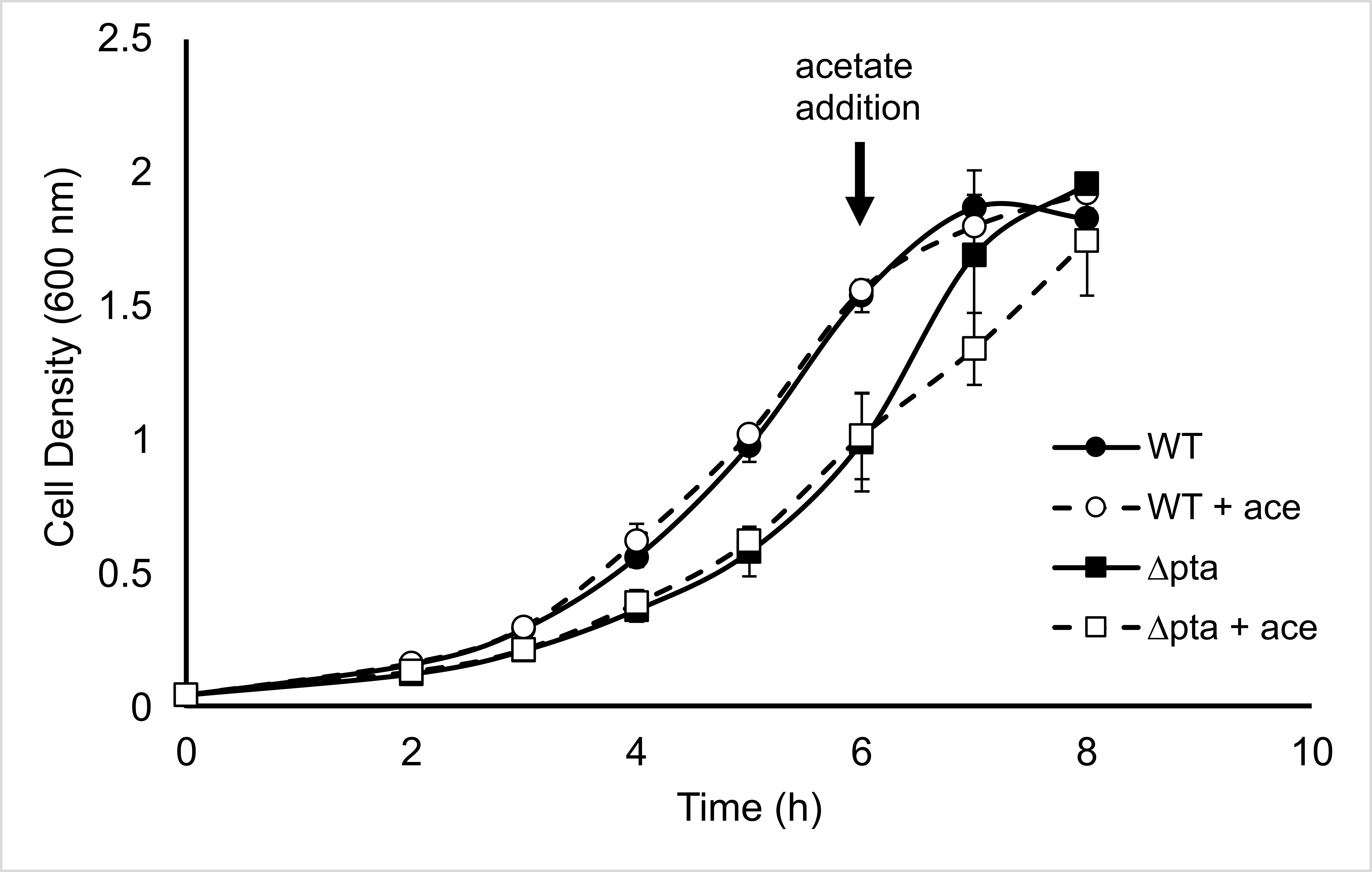

Supplement: FIG S1 [file mbio.01224-22-s0001.tif]

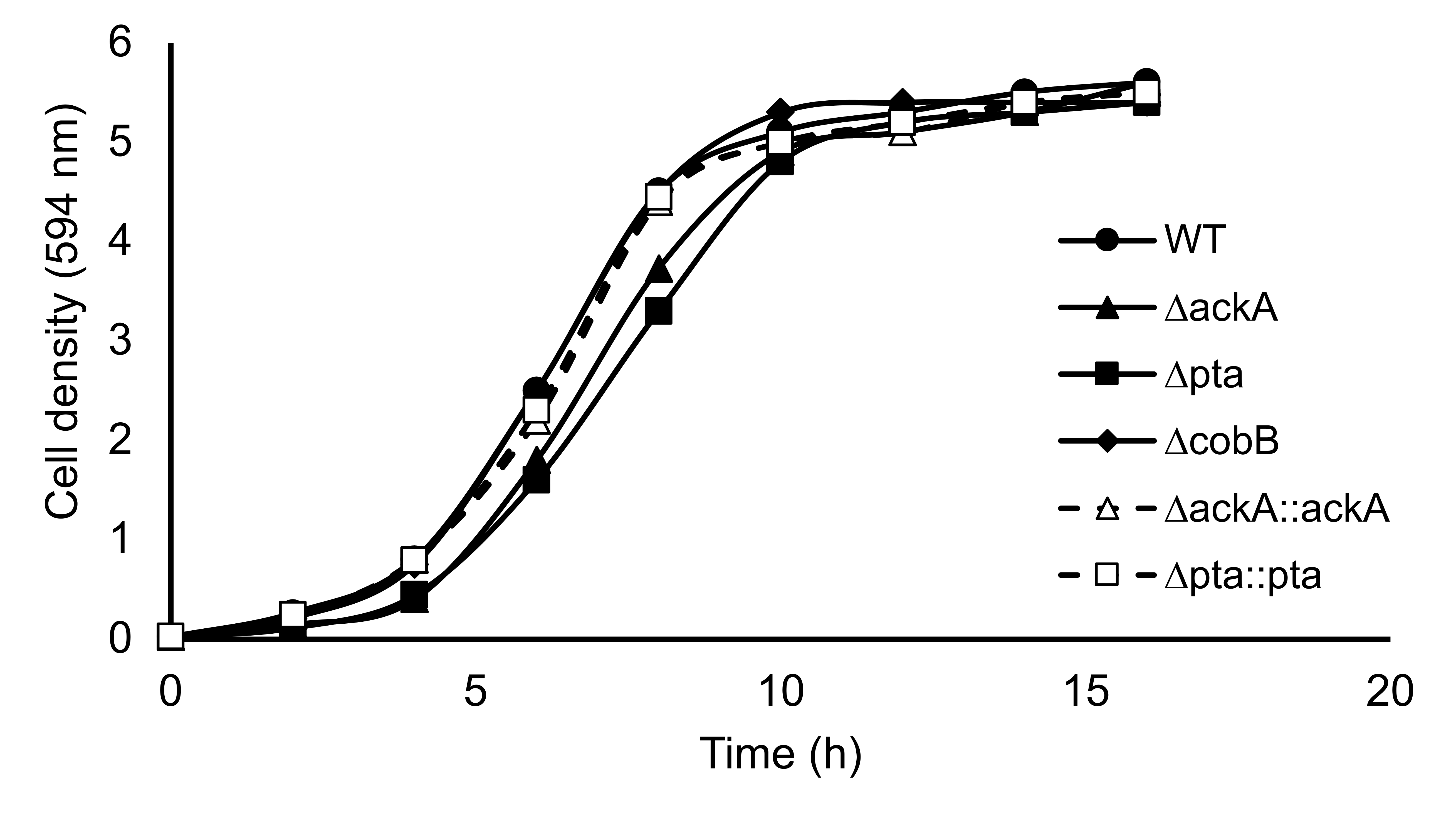

Supplement: FIG S2 [file mbio.01224-22-s0002.tif]
